# The Trials of a Hospital Physician

**Published:** 1916-12-16

**Authors:** 


### The Trials of a Hospital Physician.

It is curious how incapable some patients are of describing their symptoms or assisting the physician in his investigation into their cases. As a rule, children are the best witnesses in their own cases, and give an intelligible and straightforward account. You ask a child what is the matter with him, and he says at once, "I have hurt my arm," holding out the injured member for inspection. That tells one at once all one wants to know. But when the child's father presents himself and is asked the stereotyped question, "Well, what's the matter?" he begins something in this way: "Me and my mate got a day off, and so we thought we'd go to Epsom for a beano, so I goes to Bill Jones, what has a moke and a barrer, and 'Bill,' I says, 'how much for your moke and barrer for a day to Epsom?'" and so forth and so on.

The child's mother adopts a different attitude. She is intensely suspicious, and is not going to give herself away; and the conversation proceeds something as follows:

"Well, my good woman, what's the matter?"

"Ah! that's just what I come 'ere to find out."

"Well, but what do you complain of?"

"Oh, I don't complain. We all got to bear our troubles, and I bear mine. Gawd knows what's best for us, and it isn't for us to complain of what He sends us."

"But why do you come here?"

"Why, to be made better, o' course. What do you expect I come 'ere for? Pleasure? It ain't much pleasure to wait for an hour and a 'arf in that draughty 'all."

"Well, then, where is the pain?"

"All over me. You couldn't put a pin where I don't ache, and my poor back, well there! I feel sometimes as I shall break in two. Mrs. Brown, she says it's the rheumatics, but I don't know. I think it's the liver."

"Where is it worst?"

"Well, I don't know, I think it's when I'm at the wash-tub, but sometimes it kitches me so I almost fall down with it."

Physician (*in despair*): "Here, Mr. Jones, will you take the history of this patient?"

